# Rehabilitation Effects of Fatigue-Controlled Treadmill Training After Stroke: A Rat Model Study

**DOI:** 10.3389/fbioe.2020.590013

**Published:** 2020-11-30

**Authors:** Yuchen Xu, Yuanfa Yao, Hao Lyu, Stephanie Ng, Yingke Xu, Wai Sang Poon, Yongping Zheng, Shaomin Zhang, Xiaoling Hu

**Affiliations:** ^1^Qiushi Academy for Advanced Studies, Zhejiang University, Hangzhou, China; ^2^Key Laboratory of Biomedical Engineering of Ministry of Education, Zhejiang Provincial Key Laboratory of Cardio-Cerebral Vascular Detection Technology and Medicinal Effectiveness Appraisal, College of Biomedical Engineering & Instrument Science, Zhejiang University, Hangzhou, China; ^3^Department of Endocrinology, The Affiliated Sir Run Run Shaw Hospital, Zhejiang University School of Medicine, Hangzhou, China; ^4^Division of Neurosurgery, Department of Surgery, Prince of Wales Hospital, The Chinese University of Hong Kong, ShaTin, Hong Kong; ^5^Department of Biomedical Engineering, The Hong Kong Polytechnic University, Kowloon, Hong Kong

**Keywords:** fatigue-controlled, electromyography, training intensity, stroke, motor recovery

## Abstract

**Background:** Traditional rehabilitation with uniformed intensity would ignore individual tolerance and introduce the second injury to stroke survivors due to overloaded training. However, effective control of the training intensity of different stroke survivors is still lacking. The purpose of the study was to investigate the rehabilitative effects of electromyography (EMG)-based fatigue-controlled treadmill training on rat stroke model.

**Methods:** Sprague–Dawley rats after intracerebral hemorrhage and EMG electrode implantation surgeries were randomly distributed into three groups: the control group (CTRL, *n* = 11), forced training group (FOR-T, *n* = 11), and fatigue-controlled training group (FAT-C, *n* = 11). The rehabilitation interventions were delivered every day from day 2 to day 14 post-stroke. No training was delivered to the CTRL group. The rats in the FOR-T group were forced to run on the treadmill without rest. The fatigue level was monitored in the FAT-C group through the drop rate of EMG mean power frequency, and rest was applied to the rats when the fatigue level exceeded the moderate fatigue threshold. The speed and accumulated running duration were comparable in the FAT-C and the FOR-T groups. Daily evaluation of the motor functions was performed using the modified Neurological Severity Score. Running symmetry was investigated by the symmetry index of EMG bursts collected from both hind limbs during training. The expression level of neurofilament-light in the striatum was measured to evaluate the neuroplasticity.

**Results:** The FAT-C group showed significantly lower modified Neurological Severity Score compared with the FOR-T (*P* ≤ 0.003) and CTRL (*P* ≤ 0.003) groups. The FAT-C group showed a significant increase in the symmetry of hind limbs since day 7 (*P* = 0.000), whereas the FOR-T group did not (*P* = 0.349). The FAT-C group showed a higher concentration of neurofilament-light compared to the CTRL group (*P* = 0.005) in the unaffected striatum and the FOR-T group (*P* = 0.021) in the affected striatum.

**Conclusion:** The treadmill training with moderate fatigue level controlled was more effective in motor restoration than forced training. The fatigue-controlled physical training also demonstrated positive effects in the striatum neuroplasticity. This study indicated that protocol with individual fatigue-controlled training should be considered in both animal and clinical studies for better stroke rehabilitation.

## Introduction

Stroke is a leading cause of mortality and disability globally, resulting in substantial costs and long-term healthcare burden on both family and society (Gorelick, 2019). With the fast expansion of stroke populations worldwide and insufficiency of rehabilitation professionals in the public health, more than 50 to 60% of stroke survivors are still experiencing moderate to severe motor impairments, which significantly deteriorates their independence in daily living, even after the routine treatments (Hendricks et al., 2002). More effective post-stroke rehabilitation programs are needed to improve the effectiveness of the current treatments (Donkor, 2018).

Effective post-stroke motor restoration depends on timely physical treatments with necessary intensities, facilitating rehabilitative neuroplasticity after the lesion (Hylin et al., 2017). Both clinical and animal studies showed that post-stroke rehabilitation implemented in the subacute period introduced significantly higher neuroplasticity and better motor function recovery than those achieved in the chronic period (Yang et al., 2003; Biernaskie et al., 2004; Cumming et al., 2011). It was because physical training in the early stage after stroke, e.g., the subacute period, could more effectively induce use-dependent neural rewiring and strengthen the synapses during the spontaneous neurological recovery, compared with the later chronic stage when the neuroplastic activities tend to be stable (Yang et al., 2003; Biernaskie et al., 2004; Murphy and Corbett, 2009; Cumming et al., 2011; Bell et al., 2015). However, it also had been found that overloaded training in the early stage after stroke could inhibit functional motor recovery. For example, clinical studies found that physical practices with higher intensities in daily treatment during the subacute period post-stroke was related to worse motor outcomes with more complications compared with those who received standard training (Dromerick et al., 2009; Andrews et al., 2015). Overloaded physical training could introduce additional fatigue resulting in neurological stress that suppressed neural plasticity, motor relearning, and even enlarged brain lesion after stroke. A previous study demonstrated that fatigue induced by physical exercise could impair neural regeneration in the corticostriatal pathway, which could further deteriorate movement control and limit motor relearning in post-stroke rehabilitation (Ma et al., 2018). It was also found that the detrimental effects on the overall motor skill acquisition after fatigue could carry on to subsequent practicing days, even in the absence of fatigue (Branscheidt et al., 2019). Furthermore, in rodent models, forced training was found to increase the levels of the stress hormone, corticosterone, in serum (Sun et al., 2014), and enlarged brain lesion in the subacute stage after stroke (Kozlowski et al., 1996). Corticosterone could downregulate the concentration of the neuron survival and growth-related proteins, e.g., neurofilament-light (NFL), leading to feebler neuroplasticity (Cereseto et al., 2006).

Fatigue level is an essential parameter that should be managed for an optimized motor outcome in post-stroke rehabilitation. However, the training intensity is usually uniformly administrated to a batch of patients in the traditional physio- and occupational treatments, e.g., grouped training with the same intensity, for an easy management with limited professional manpower. On the other hand, patients might also force themselves to complete the assigned training intensities, even if overloaded, with the expectation of promised motor improvements. The fatigue tolerance of individual patients could be diverse to the same training intensity. There is a lack of regulation on the individualized fatigue level during the physical training after stroke in the current clinical practices.

Although rat models with subacute stroke have been investigated on the effects of varied running intensities on motor outcomes, the fatigue levels based training has not been regulated explicitly in the previous studies (Sun et al., 2014; Chen et al., 2015). For example, Sun et al. (2014) demonstrated that running schemes on a treadmill with gradually increased speeds along with the motor recovery progress and adaptive speeds with intermittent stops in a wheel could achieve better motor outcomes than those with the schemes of constant running speed throughout the training (Chen et al., 2015). The results in these studies implied that physical training with a fatigue level adaptive to the motor recovery progress, or the tolerance of individual rats, could achieve more effective motor recovery in early post-stroke rehabilitation.

The individual fatigue level during physical training could be continuously monitored by electromyography (EMG) on the rehabilitation task-related main contracting muscles (Dobkin, 2008). A drop of mean power frequency (MPF) in EMG could be captured in a fatiguing muscle, which had been used as a biomarker in sports medicine (Cifrek et al., 2009; Li et al., 2011; Wang et al., 2018). However, using EMG MPF to regulate the fatigue level in post-stroke physical rehabilitation has not been carried out previously.

In this work, we established a treadmill running platform with fatigue level controlled by monitoring the EMG MPF during running for individual rats in the subacute stage after stroke, with the purpose of avoiding overloaded physical training. We hypothesized that the fatigue-controlled treadmill training would achieve better rehabilitation effects than those administrated with forced and continuous running with a constant speed, which simulated the batched treatment with uniform intensity. The rehabilitation effects were investigated by behavioral assessments, EMG biomarkers, and NFL expression levels in the brain tissues.

## Materials and Methods

**Figure 1:**
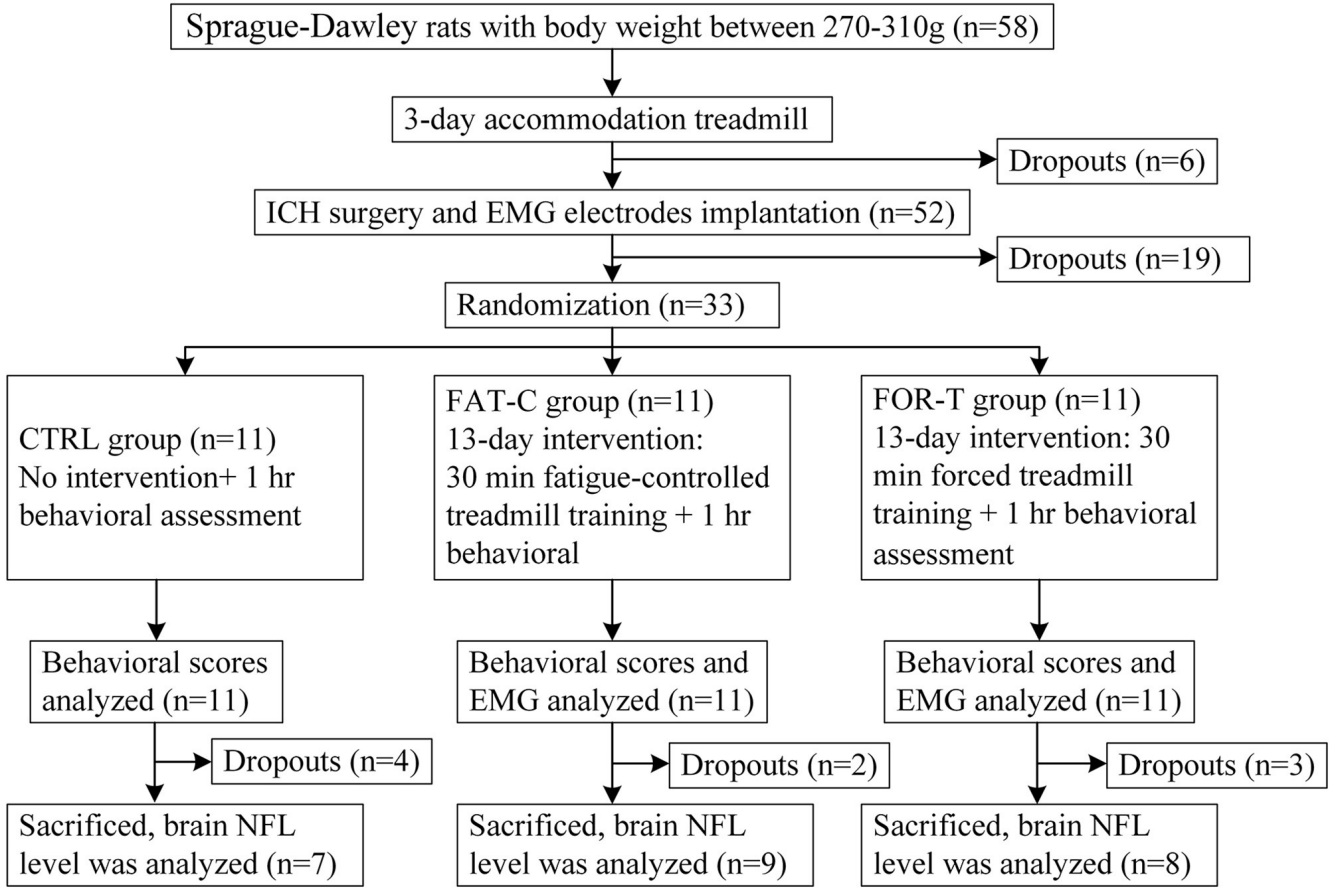
Consort flowchart of the experimental design.

In this study, a custom-made treadmill training system with the control of fatigue level based on EMG was designed as the experimental platform for post-stroke training. The rats with intracerebral hemorrhage (ICH) received fatigue-controlled or forced treadmill training in this system to investigate the effects of fatigue-controlled training on motor function recovery. The design of the experiment is shown in Figure 1. All rats performed a 3 day treadmill accommodation running, followed by surgeries of ICH and EMG electrode implantation. Two days after the operations, the rats were divided into three groups randomly and received fatigue-controlled, forced, or no training during the subacute period (day 2 to day 14 post-stroke) (Forghani et al., 2015). The recovery effects were evaluated by behavioral tests, EMG signal markers, and brain tissue Western blot analysis.

### Animal Preparation

Fifty-eight Sprague–Dawley adult rats weighted from 270 to 310 g were included in this study. They were housed in a 12/12 h light/dark vivarium with *ad libitum* access to food and water except for experimental periods. All animals went through a 30 min treadmill running at the speed of 16 m/min per day for 3 consecutive days to get familiar with running on the treadmill (Rezaei et al., 2017). The length of the runway was 50 cm, and the treadmill controller would show the accumulative running duration (ZH-PT animal experiment treadmill, Anhui Zhenghua Biological Instrument, China). Rats with occasional stops during running would receive gentle nudges to help them keep running (Ke et al., 2011). If the rat could not keep running in the middle of the track for more than 15 min on the third day, it would be excluded from the study. Six rats were excluded after the 3 day adaptation running.

### Surgeries of Intracerebral Hemorrhage and Electromyography Electrodes Implantation

Fifty-two rats passed through the running adaptation and received the surgeries of ICH and EMG electrode implantation. The rat was anesthetized by propofol (1.2 mg/kg, intraperitoneally, and 0.02 mg/kg subsequently) to achieve a 3 h around narcotism for the ICH surgery and the consecutive EMG electrode implantation.

#### Intracerebral Hemorrhage Surgery

**Figure 2:**
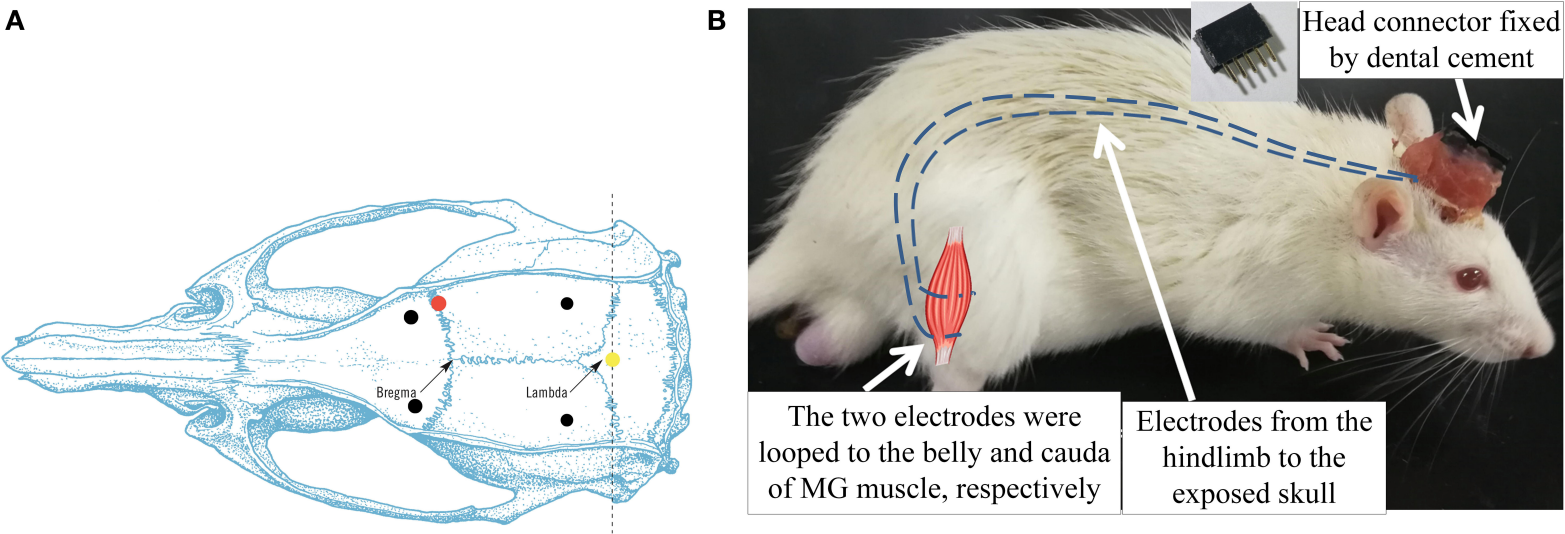
**(A)** Dorsal skull surface of rat showed the locations of the fixed screws (black dots), common ground (yellow dot), and injection hole (red dot). **(B)** Illustration of intramuscular electrode implantation on MG and the head connector. Dash lines represented the EMG electrodes.

ICH surgery is a common model adopted for investigations on post-stroke motor recovery (Kelly et al., 2003; Park et al., 2010; Bai et al., 2012). It disrupts the blood vessels of the basal lamina and leads to bleeding and contralesional hemiplegia in rats (Chesney et al., 1995). The surgery was guided by a stereotaxic system, and the ICH lesion was introduced by intrastriatal administration of bacterial collagenase according to the method in MacLellan et al. (2006), with the anatomical illustration shown in Figure 2A. The brain slices in the previous study showed similar bleeding lesion sizes according to the surgical protocol (Liu et al., 2013). During the operation, the rat was secured prone onto a stereotaxic apparatus (68005, RWD Life Science, China) with a heating pad to keep body temperature maintained at 36–37°C (Wangfischer, 2009). After making an incision over the scalp, the striatum in the right hemisphere was located through stereotaxic coordinates: 0.2 mm anterior, 3.0 mm lateral (Figure 2A, red dot), and 6.0 mm ventral to the Bregma. A 1 mm diameter borehole was drilled (Figure 2A, red dot) by trephine (78001, RWD Life Science, China), and the 26 gauge, 5 μl syringe (Hamilton syringe 700, Hamilton, USA) was inserted into the striatum. Type IV collagenase (1.2 μl, 0.25 U in 1 μl NaCl 0.9%, C5138, Sigma, USA) was infused using a micro-infusion pump (Micro 4, World Precision Instruments, Inc., USA) for 5 min. The syringe was made to remain in place for 10 min to avoid backflow and subsequently withdrawn slowly (Liu et al., 2013). Then, the hole was sealed by medical glue. The wound on the scalp was left for the following EMG electrode implantation.

#### Electromyography Electrode Implantation

After the ICH surgery, the intramuscular electrodes were implanted on the AF and UN medial gastrocnemius muscles (MG) (Li et al., 2013). A differential electrode configuration was adopted for the measurement of EMG activity of the target muscles. A skin incision was made on the dorsal side of the calf to expose the MG. The insulation was stripped off from the end of the Teflon-coated stainless steel wires (AS632, Cooner Wire, USA) by 2 cm (Li et al., 2011). After separating the skin from the muscular layer through blunt dissection, the two wires were inserted and looped around the belly and cauda of the MG (Barroso et al., 2019) (Figure 2B). The four implanted electrodes were tunneled subcutaneously from both hind limbs to the exposed skull and soldered with a five-pin head connector (Figure 2B). The fifth pin was connected to the common ground screw on the skull (0.0 mm anterior and 0.0 mm lateral to Lambda, Figure 2A, yellow dot) through a silver wire. Four screws were anchored firmly to the skull (2.0 mm anterior and ±2.0 mm lateral to the Bregma, 2.0 mm anterior and ±3.0 mm lateral to the Lambda, Figure 2A, black dots) as a foundation of the head connector. The connector was fixed on the skull by screws with dental cement. After the suturing of the wounds, the rat was put in a warm box [45 cm (length) × 35 cm (width) × 30 cm (height)] to maintain the body temperature at 36–37° (Wangfischer, 2009) with *ad libitum* access to food and water till waking up. Fifteen rats dropped out within the first 2 days after ICH surgery, mainly due to severe cerebral hemorrhage.

### Randomization

Two days (48 h) after the ICH and EMG electrode implantation surgeries, 37 rats survived. Thirty-three rats with modified neurological severity score (mNSS) > 6 on day 2 post-stroke were recruited to guarantee the comparable degree of motor impairments. They were distributed randomly into three groups, i.e., the CTRL (*n* = 11), the FAT-C (*n* = 11), and the FOR-T groups (*n* = 11).

### Rehabilitation Intervention

#### Fatigue-Controlled System

**Figure 3:**
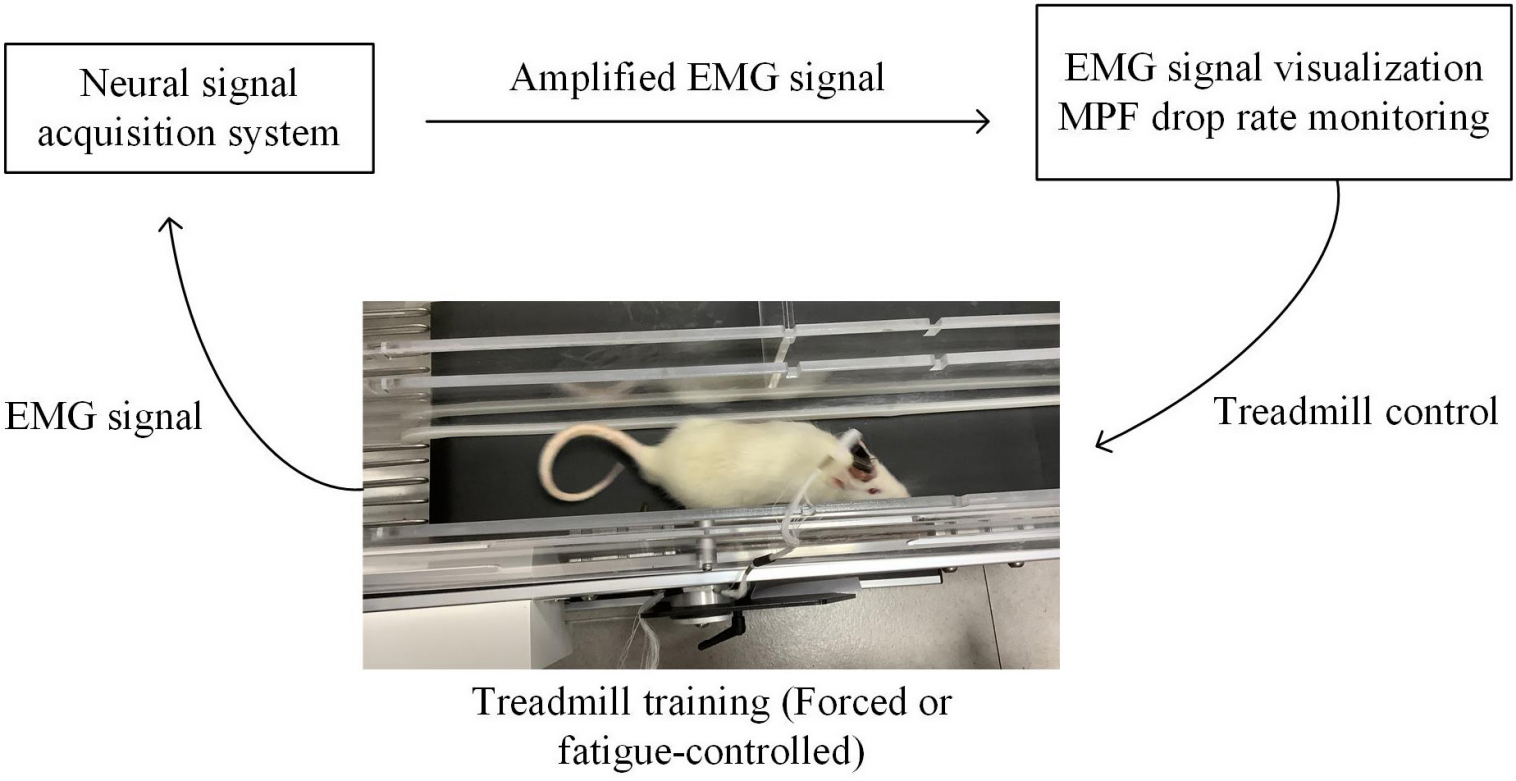
Fatigue-controlled experimental platform. Rat was running on the treadmill; its EMG signal was acquired and amplified by a neural data acquisition system (Plexon Inc., USA) and transmitted to the computer. EMG signal was real-time visualized on the computer screen. The Matlab GUI-based fatigue-controlled software calculated MPF drop rate during treadmill training, and the on/off state of the treadmill was controlled by the MPF drop rate.

In this study, we integrated the treadmill with an EMG analysis system based on a customized MATLAB graphical user interface (GUI) (Matlab, 2016a) to facilitate the fatigue-controlled treadmill training. The structure diagram of the training system is illustrated in Figure 3, including three parts: (1) a multitrack treadmill, (2) EMG amplification and acquisition system, and (3) an online signal processing module with GUI.

**Figure 4:**
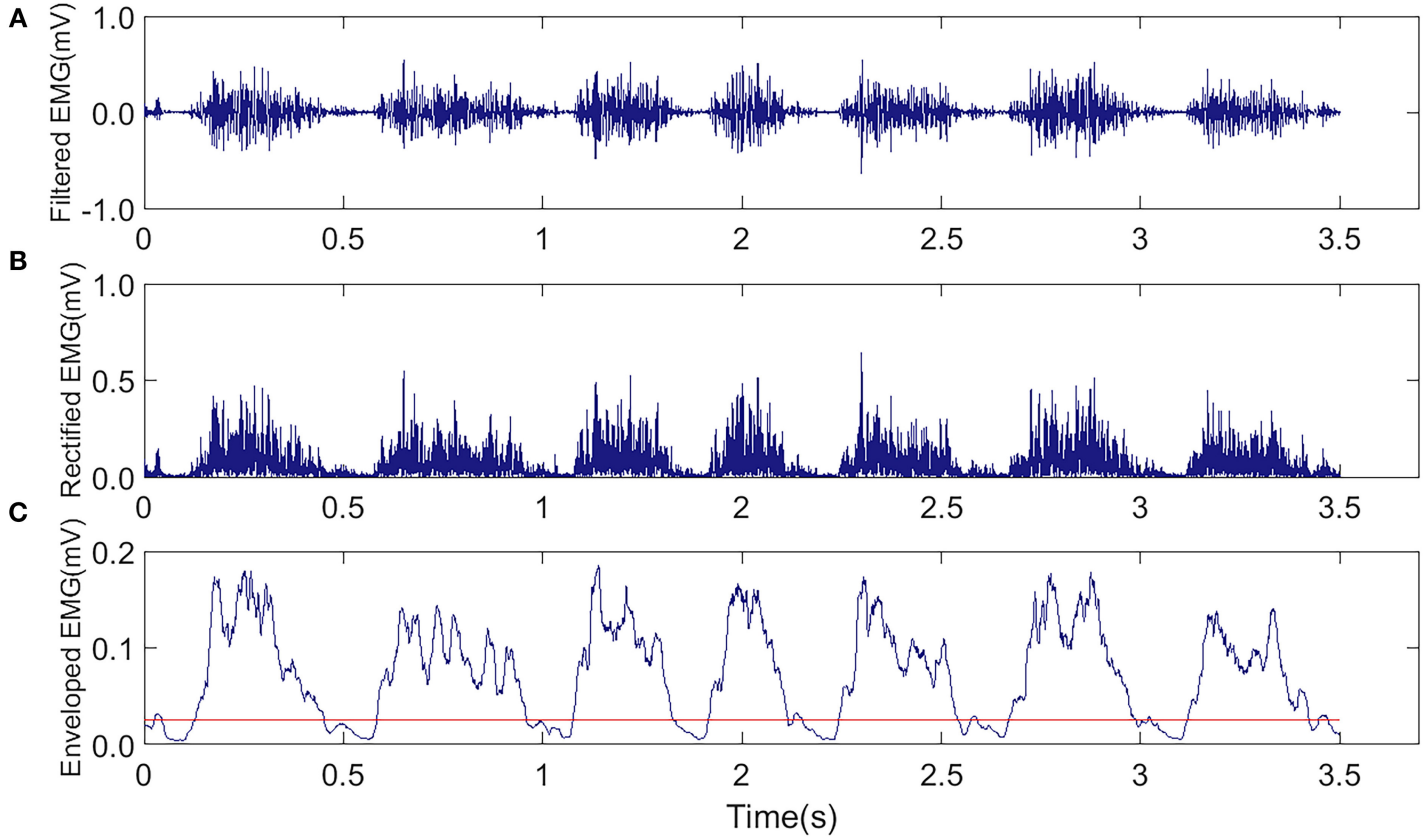
Representative EMG trials were captured from the MG muscle in the AF side of a rat during the treadmill running in the FAT-C group. **(A)** Raw EMG after band-pass filtering (60–2,000 Hz). **(B)** Rectified EMG. **(C)** Enveloped EMG, the red line was the threshold of EMG bursts.

The EMG signals from the MG muscles in both hind limbs were amplified with a gain of 1,750 by a neural signal acquisition system (OmniPlex Neural Signal Acquisition System, Plexon Inc., USA), with a sampling rate of 40 kHz and band-pass filtered with a fourth-order Butterworth filter (60–2,000 Hz). Figure 4A shows the representative EMG trials during the treadmill running. The recorded EMG signals went through visual inspection for removing motion artifacts. Then, the filtered raw EMG signals were further rectified (Figure 4B). Moving average window with a length of 25 ms was applied to obtain the envelope of the rectified EMG (Figure 4C). The onset and end of a burst duration related to muscle contraction were identified by the threshold (Figure 4C, red line), which was defined as the mean value of the enveloped EMG plus 1.5 standard deviations during the resting period (Li et al., 2013).

The EMG signals were displayed in real-time for monitoring the quality and imported to the computer for storage through the MATLAB GUI via the Plexon Control and Server software (OmniPlexVersion 1.16.1, Plexon Inc., USA) supplied by MATLAB Online Client Development Kit (Plexon Inc., USA). The GUI-based software also visualized the MPF drop rate by calculating the following:

(1) $$\begin{array}{l} MPF=\;\frac{\int_{0}^{\infty }f•s\left(f\right)df}{\int_{0}^{\infty }s\left(f\right)df} \end{array}$$

where *s(f)* was the power spectrum density of the EMG signal. The *MPF* value was calculated every 4 s during treadmill training, which was sufficient and stable to represent the change of muscle fatigue.

The *MPF drop rate* was defined as (Li et al., 2011),

(2) $$\begin{array}{l} MPF\;drop\;rate=\;\frac{baselineMPF-runningMPF}{baselineMPF}\cdot 100\% \end{array}$$

where the *baseline MPF* was the average *MPF* values in the first 20 s in the 30 min treadmill training. The *running MPF* was the *MPF* values calculated based on the EMG of the target hind limb during treadmill training. Once the *MPF drop rate* exceeded a preset threshold, the software triggered an audio alarm.

#### Training Protocol

Different rehabilitation interventions were delivered to the three groups from day 2 to day 14 post-stroke. No rehabilitation training was conducted for the CTRL group. For the FOR-T group, the rats were forced to run on the treadmill at a speed of 16 m/min with a duration of 30 min each day without rest (Sun et al., 2014; Chen et al., 2015; Rezaei et al., 2017). If the rats stayed at the end of the treadmill track and failed to catch up with the treadmill, the experimenters would give gentle pushes in the back of rats to force them to continue the running.

For the FAT-C group, the MPF drop rate of the AF hind limb was monitored by the fatigue-controlled system during the treadmill training. Once the MPF drop rate exceeded the threshold, the treadmill would be stopped, and a 3 min rest was applied to the rat, as 3 min rest was enough to ease the fatigue (Carroll et al., 2017). The speed of the treadmill was 16 m/min, and the accumulated running duration was 30 min/day for the FAT-C group. In this work, the fatigue level of the AF hind limb was adopted, with the main purpose to monitor the usage of the paretic hind limb and to minimize the compensatory effects in the UN hind limb (Pin-Barre and Laurin, 2015). A moderate training intensity achieved better motor function recovery in post-stroke rehabilitation, and the MG MPF drop rate was around 11% during moderate treadmill training (Li et al., 2013), so an 11% MPF drop rate was used as the fatigue level threshold of FAT-C group. Three rats (FOR-T: *n* = 2, FAT-C: *n* = 1) dropped out because of EMG electrodes broken during treadmill training, and two rats in the CTRL group dropped out due to severe hemorrhage during day 2 to day 5.

Before collecting EMG, we first checked the noise level of the EMG in both the time and frequency domains to ensure the close connection between the recording headstage and the electrode connector on the head of a rat during training. We also fastened the connection with tape to minimize the motion artifacts.

### Evaluation

In this study, the mNSS was applied to evaluate the effects of fatigue-controlled training on post-stroke motor function recovery. The symmetry index (SI) of hind limbs based on EMG was used to evaluate the balance during running. The expression of protein NFL in the striatum was measured to explore the degree of axonal plasticity after the 13 day rehabilitative interventions.

#### Modified Neurological Severity Score

The mNSS has been used to assess the neurologic deficits of stroke rats (Schaar et al., 2010; Liu et al., 2013), based on behavioral tests. Assessments were conducted by an experimenter blinded to the training protocol and group information of the rats from day 2 to day 14 before daily rehabilitation intervention. Neurologic function was graded on a scale of 0–18, with 0 indicating normal neurologic function and 18 maximum functional deficits (Liu et al., 2013). The mNSS was further decomposed into motor (muscle status and abnormal movement, maximum 6), sensory (visual, tactile and proprioceptive, maximum 2), beam balance (maximum 6), and reflexes/abnormal movements (maximum 4) (Schaar et al., 2010).

#### Symmetry Index

The SI was used to quantify the balance function between the two hind limbs during the running on the treadmill by EMG (Li et al., 2011). It was calculated by the burst duration of the enveloped EMG on the AF and UN side muscles during treadmill training:

(3) $$\begin{array}{l} SI=\frac{{BurstDuration}_{AF}-{BurstDuration}_{UN}}{{BurstDuration}_{AF}+{BurstDuration}_{UN}}\;\cdot 200\% \end{array}$$

When SI was zero, it represented a perfect balance between the hind limbs. In contrast, a negative SI that represented the UN hind limb showed a longer burst duration than the AF hind limb, vice versa.

#### Expression Level of Neurofilament-Light in the Striatum

The rehabilitative effects were also evaluated by the level of neurofilaments in the brain tissue after the treadmill training. Neurofilaments are elastic and fibrous proteins (Mages et al., 2018) and are highly related to regeneration and plasticity in axons (Yuan et al., 2012; Bragina and Conti, 2018). In this study, the expression level of NFL in the striatum was evaluated as a biomarker for the axonal plasticity, as the NFL was the backbone of the neurofilaments (Gaetani et al., 2019) and the striatum was the lesion area after ICH (Joseph et al., 2016).

The rats were killed at day 14 post-stroke. The tissue preparation and Western blot analysis were conducted according to the protocol in the previous study (Mages et al., 2018). Four rats dropped out due to blur boundaries of the striatum (CTRL: *n* = 2, FOR-T: *n* = 1, and FAT-C: *n* = 1). Twenty-four rats were used in this test (CTRL: *n* = 7, FOR-T: *n* = 8, and FAT-C: *n* = 9). After transcardial perfusion with saline, the brain tissue was removed. We extracted the striatum from both the hemispheres. These specimens were homogenized and lysed in lysis solution (P0013B, radioimmunoprecipitation assay lysis buffer, Beyotime, China) with protease inhibitor cocktail (04693132001, complete inhibitor cocktail, Roche, USA) on ice. The lysate was then centrifugated at 13,000 revolutions per minute at 4°C for 10 min. The supernatant was mixed with loading buffer and denatured at 95°C for 5 min. The proteins were separated through 5% spacer gel and 10% separation gel at 100 and 120 V, respectively. Then proteins were transferred to the polyvinylidene fluoride membrane at 200 mA for 2 h. After that, the membrane was blocked by bovine serum albumin for 30 min and incubated with primary antibodies for NFL and glyceraldehyde-3-phosphate dehydrogenase (GAPDH) (NFL: 1:1,000, C28E10; GAPDH: 1:1,000, D16H11, Cell Signaling Technology, USA) at 4°C overnight and incubated with secondary antibody for 2 h at room temperature. The proteins were detected by Tanon software (TanonImage, V1.00, China) using the enhanced chemiluminescent kit (UB279013, Thermo Scientific, USA). The relative protein expression was analyzed with Image J software (1.43u, National Institutes of Health, USA) and controlled with the corresponding expression level of GAPDH.

### Statistical Analysis

The normality test was performed on all data samples by the Kolmogorov–Smirnov test with a significant level of 0.05, and all data samples had normal distributions (*P* > 0.05). Differences in the MPF drop rate between the FOR-T and FAT-C groups were evaluated using a two-way analysis of variance (ANOVA). The intragroup changes of MPF drop rate at different time points and intergroup differences of MPF drop rate at the same time point were evaluated using one-way ANOVA followed by *post hoc* Bonferroni tests. Behavioral scores among the three groups were evaluated by two-way analysis of covariance (ANCOVA). The intragroup changes of behavioral scores at different time points and intergroup differences of behavioral scores at the same time point were evaluated using one-way ANOVA and one-way ANCOVA, respectively, followed by *post hoc* Bonferroni tests. The covariate was the behavioral score on day 2 post-stroke to exclude the possible deviations of initial motor impairment among the groups. Two-way ANCOVA evaluated the differences of SI between the FOR-T and FAT-C groups, and the intragroup differences were evaluated through one-way ANOVA followed by *post hoc* Bonferroni tests. The covariate was the SI on day 2 post-stroke to exclude the possible deviations of the initial ability of hind limb balance between the groups. The protein level of NFL among the three groups was investigated by one-way ANOVA followed by *post hoc* Bonferroni tests. *P* < 0.05 was adopted as a statistically significant level in this study. Significant levels of *P*<0.005 and <0.001 were also indicated. All statistical analyses were performed using SPSS (version 20, IBM, USA).

## Results

### Electromyography Mean Power Frequency Drop Rate During Treadmill Training

**Figure 5:**
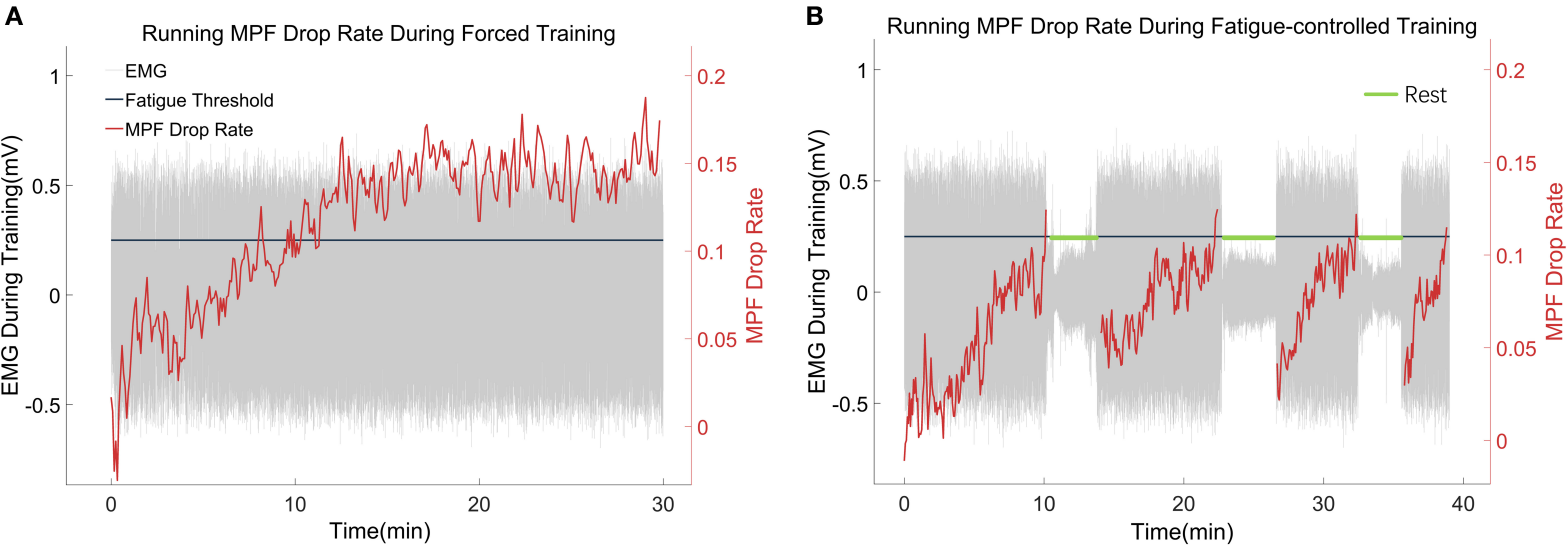
**(A,B)** Representative trials of the MPF drop rate during treadmill training in the FOR-T group and FAT-C groups. The red lines were real-time MPF drop rates. The gray parts were EMG thumbnails, and the amplitude around ±0.5 mV stood for scuttle, while the amplitude around 0 mV represented the 3 min rest. The black line was the MPF threshold for the FAT-C group.

**Figure 6:**
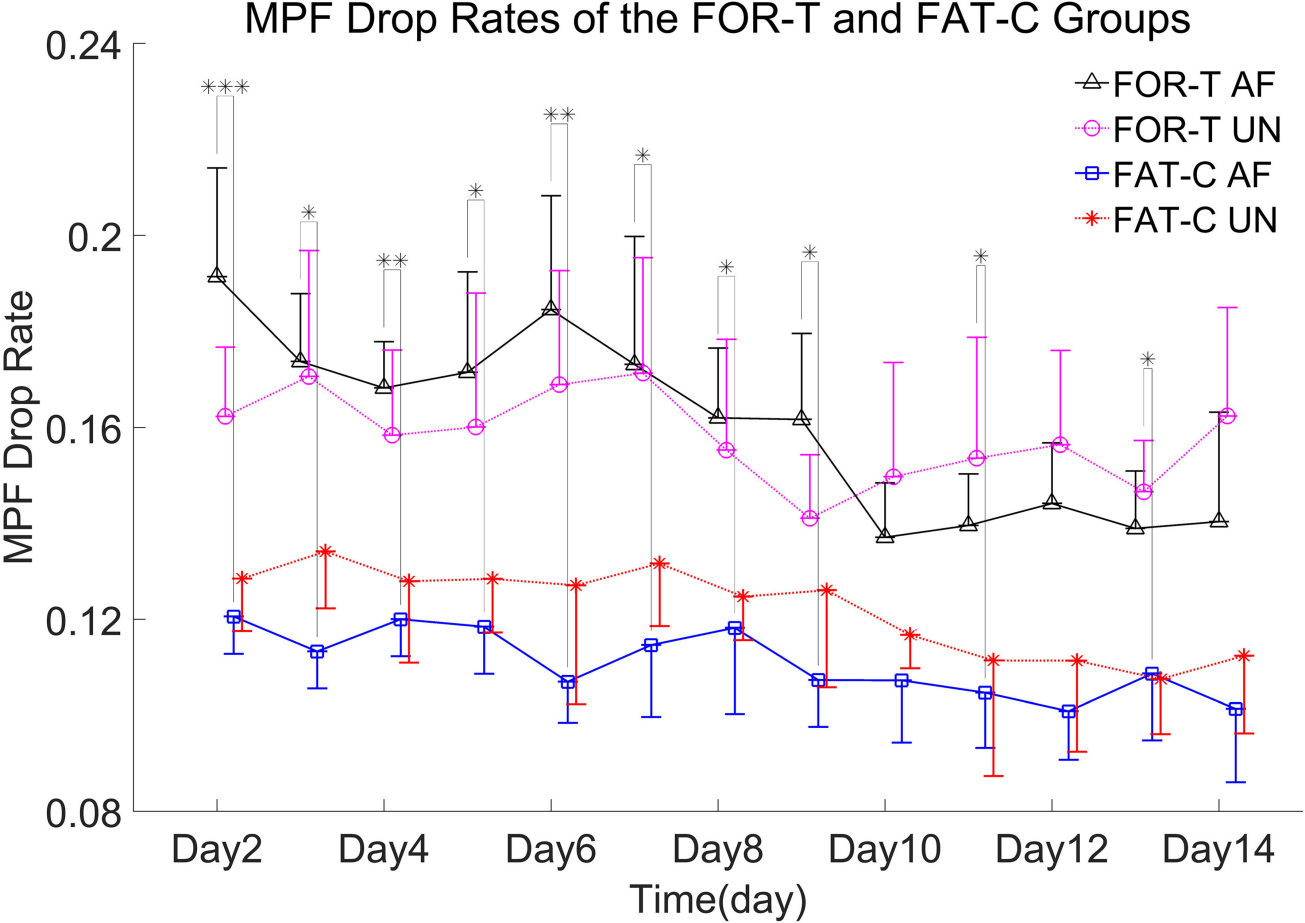
MPF drop rates of the AF/UN hind limbs in the FOR-T and FAT-C groups from day 2 to day 14 post-stroke. Values were represented by mean and 0.5*SD (error bars) at each time point. Significant differences between the MPF drop rates in the AF hind limb of FAT-C and FOR-T groups were indicated by “*”. Significant levels were indicated as 1 superscript for <0.05, 2 superscripts for <0.005, and 3 superscripts for <0.001.

In this work, the fatigue level was monitored through the EMG MPF drop rate during treadmill training in both the FOR-T and FAT-C groups. The representative trials on the MPF drop rates paired with EMG signals obtained in the FOR-T and FAT-C groups are shown in Figures 5A,B, respectively. Figure 6 shows the daily MPF drop rates of both the hind limbs in the FOR-T and FAT-C groups. Table 1 summarizes the mean values and 95% confidence intervals of the MPF drop rates in Figure 6 and the results of two-way ANOVA with effect sizes (EFs), concerning the factors of timepoint and group. The probabilities for the MPF drop rate varying over the time in each group and those for the intergroup comparison on each different timepoints analyzed by one-way ANOVA are also included in Table 1.

**Table 1:** Comparison of the MPF drop rate with respect to the independent factors of the time point and group.

**Time point (Day)**	**MPF drop rate, mean (95% confidence interval)**				**One-way ANOVA, *P*-value (EF)**	**Two-way ANOVA**, ***P*****-value (EF)**		
	**FAT-C**	**FOR-T**	**FAT-C**	**FOR-T**		**Time point**	**Group**	Time point*Group
	**AF**		**UN**					
Day 2	0.121 (0.099–0.142)	0.191 (0.168–0.214)	0.128 (0.105–0.152)	0.162 (0.139–0.185)	0.000*** (0.515)	0.006* (0.070)	0.006* (0.295)	0.990 (0.047)
Day 3	0.113 (0.084–0.143)	0.174 (0.146–0.201)	0.134 (0.105–0.164)	0.171 (0.145–0.196)	0.011* (0.376)			
Day 4	0.120 (0.099–0.141)	0.168 (0.151–0.186)	0.128 (0.107–0.149)	0.158 (0.139–0.178)	0.003** (0.391)			
Day 5	0.118 (0.091–0.146)	0.171 (0.147–0.196)	0.128 (0.101–0.156)	0.160 (0.133–0.188)	0.020* (0.275)			
Day 6	0.107 (0.076–0.138)	0.193 (0.162–0.224)	0.127 (0.095–0.160)	0.169 (0.138–0.200)	0.001** (0.391)			
Day 7	0.115 (0.086–0.143)	0.173 (0.147–0.199)	0.132 (0.103–0.160)	0.171 (0.144–0.198)	0.008* (0.285)			
Day 8	0.118 (0.095–0.141)	0.162 (0.140–0.184)	0.125 (0.102–0.148)	0.155 (0.132–0.178)	0.017* (0.262)			
Day 9	0.107 (0.086–0.129)	0.162 (0.140–0.183)	0.126 (0.105–0.148)	0.141 (0.120–0.163)	0.007* (0.309)			
Day 10	0.107 (0.086–0.129)	0.137 (0.118–0.156)	0.117 (0.095–0.138)	0.150 (0.13–0.168)	0.018* (0.237)			
Day 11	0.105 (0.077–0.132)	0.140 (0.115–0.164)	0.111 (0.084–0.139)	0.154 (0.128–0.179)	0.037* (0.236)			
Day 12	0.101 (0.078–0.124)	0.144 (0.123–0.166)	0.111 (0.088–0.134)	0.156 (0.135–0.178)	0.003** (0.368)			
Day 13	0.109 (0.091–0.126)	0.139 (0.122–0.156)	0.108 (0.090–0.125)	0.147 (0.124–0.169)	0.009* (0.357)			
Day 14	0.112 (0.070–0.153)	0.140 (0.107–0.174)	0.112 (0.079–0.146)	0.162 (0.133–0.192)	0.097 (0.265)			
One-way ANOVA, *P*-value (EF)	0.844 (0.074)	0.005* (0.227)	0.843 (0.074)	0.969 (0.044)				

Differences with statistical significance are marked with “*” (two-way ANOVA, one-way ANOVA).). Significant levels were indicated as 1 superscript for <0.05, 2 superscripts for <0.005, and 3 superscripts for <0.001.

In the FOR-T group, the MPF drop rate kept increasing from the beginning of the running and exceeded 11% of the initial MPF value after about 10 min. The MPF drop rate reached a plateau and kept around 18% till the end, as shown in Figure 5A. On the other hand, the rats in the FAT-C group would take a 3 min rest once their MPF drop rate exceeded 11% and then continue the running. The MPF drop rate was reduced after the intermittent rest and kept increasing after training started again, as shown in Figure 5B. Figure 6 demonstrated that the MPF drop rates varied significantly with respect to the time point and group factors (two-way ANOVA, time point: *P* = 0.006, EF = 0.070, group: *P* = 0.006, EF = 0.295, Table 1). For the FOR-T AF hind limb group, a significant reduction in the MPF drop rate was observed over the time points (one-way ANOVA, *P* = 0.005, EF = 0.227, Table 1). There was no significant MPF drop rate variation in other groups. Significant MPF drop rate differences were found from day 2 to day 13 (one-way ANOVAs, *P* ≤ 0.037, EF ≥ 0.236, Table 1) among the AF and UN hind limbs of the FAT-C and FOR-T groups. The *post hoc* results indicated that the FOR-T AF group demonstrated a significantly higher MPF drop rate compared with the FAT-C AF group from day 2 to day 9 (one-way ANOVAs, *P* ≤ 0.02, EF ≥ 0.262, Bonferroni *post hoc* tests *P* ≤ 0.048, Table 1). In contrast, on day 10 and day 12, a significantly higher MPF drop rate was found in the FOR-T UN group compared with that in the FAT-C AF group (one-way ANOVAs, *P* ≤ 0.018, EF ≥ 0.237, Bonferroni *post hoc* tests *P* ≤ 0.025, Table 1).

### Behavioral Scores

**Figure 7:**
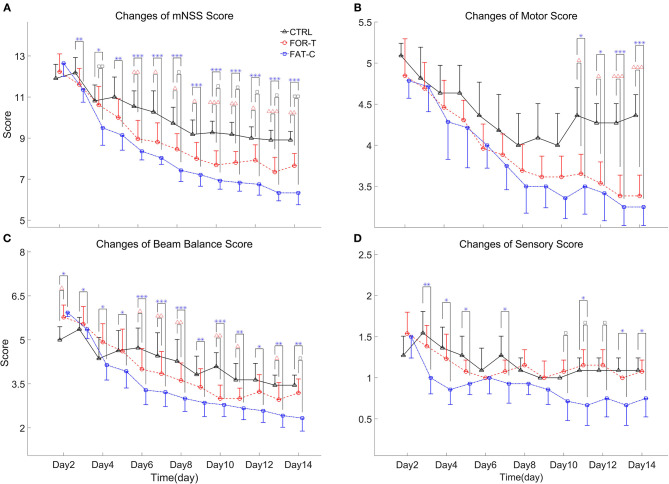
Full mNSS **(A)** and the sub-mNSS on the motor **(B)**, beam balance **(C)**, and sensory **(D)** scores for CTRL, FOR-T, and FAT-C groups from day 2 to day 14 post-stroke were presented as mean value with 0.5*SD (error bar). Significant differences between CTRL and FOR-T groups were indicated by “Δ” and the significant differences between the CTRL and the FAT-C groups were indicated by “*”. Significant differences between the FOR-T and the FAT-C groups were indicated by “□”. Significant levels were indicated as 1 superscript for <0.05, 2 superscripts for <0.005, and 3 superscripts for <0.001.

The behavioral tests were conducted from day 2 to day 14 post-stroke. In this study, ICH resulted in sensorimotor impairments captured by the motor, sensory, and beam balance assessments in the mNSS. The comparisons on the behavior scores of the CTRL, FOR-T, and FAT-C groups during the rehabilitation are shown in Figure 7. In relation to Figure 7, the detailed probabilities of the comparisons by two-way ANCOVA on the time point and group factors, one-way ANOVA in each group over the timepoint, and one-way ANCOVA of different groups at the same time point are summarized in Table 2.

**Table 2:** Comparison of behavior scores with respect to the independent factors of the timepoint and group, behavior scores on day 2 as covariate.

	**One-way ANCOVA**, ***P*****-value (EF)**											
**Behavior test**	**mNSS**			**Motor**			**Beam Balance**			**Sensory**		
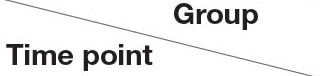	**CTRL**	**FOR-T**	**FAT-C**	**CTRL**	**FOR-T**	**FAT-C**	**CTRL**	**FOR-T**	**FAT-C**	**CTRL**	**FOR-T**	**FAT-C**
Day 3	0.002**(0.305)			0.937(0.004)			0.023*(0.2)			0.003**(0.286)		
Day 4	0.003**(0.286)			0.738(0.018)			0.022*(0.201)			0.021*(0.203)		
Day 5	0.001**(0.323)			0.531(0.037)			0.007*(0.253)			0.017*(0.213)		
Day 6	0.000***(0.498)			0.464(0.044)			0.000***(0.438)			0.593(0.030)		
Day 7	0.000***(0.413)			0.437(0.047)			0.000***(0.452)			0.034*(0.181)		
Day 8	0.000***(0.517)			0.314(0.066)			0.000***(0.463)			0.113(0.120)		
Day 9	0.000***(0.430)			0.124(0.115)			0.006*(0.262)			0.386(0.054)		
Day 10	0.000***(0.587)			0.081(0.137)			0.000***(0.389)			0.018*(0.209)		
Day 11	0.000***(0.560)			0.005*(0.282)			0.001**(0.339)			0.007*(0.266)		
Day 12	0.000***(0.501)			0.004**(0.295)			0.008*(0.26)			0.027*(0.203)		
Day 13	0.000***(0.651)			0.000***(0.464)			0.001**(0.342)			0.008*(0.258)		
Day 14	0.000***(0.641)			0.000***(0.501)			0.002**(0.334)			0.023*(0.210)		
One-way ANOVA, *P*-value (EF)	0.000*** (0.433)	0.000*** (0.612)	0.000*** (0.692)	0.120 (0.126)	0.000*** (0.416)	0.000*** (0.365)	0.000*** (0.315)	0.000*** (0.417)	0.000*** (0.486)	0.007* (0.188)	0.143 (0.103)	0.304 (0.081)
Two-way ANCOVA, *P*-value (EF)	**Time point**											
	0.000***(0.576)			0.000***(0.271)			0.000***(0.387)			0.000***(0.084)		
	**Group**											
	0.000***(0.456)			0.000***(0.118)			0.000***(0.308)			0.000***(0.166)		
	Time point*Group											
	0.589(0.046)			0.535(0.048)			0.49(0.05)			0.722(0.041)		

Differences with statistical significance are marked with “*” (two-way ANCOVA, one-way ANCOVA, one-way ANOVA). Significant levels were indicated as 1 superscript for <0.05, 2 superscripts for <0.005, 3 and superscripts for <0.001.

#### Overall Modified Neurological Severity Score

Figure 7A shows the variations of the overall mNSS for the CTRL, FOR-T, and FAT-C groups over the time points. Two-way ANCOVA suggested that the overall mNSS varied significantly with respect to the time point and group factors (two-way ANCOVA, timepoint: *P* = 0.000, EF = 0.576, group: *P* = 0.000, EF = 0.456, Table 2). The CTRL group showed a significant reduction in mNSS since day 7 (one-way ANOVA, *P* = 0.000, EF = 0.433, Bonferroni *post hoc* test *P* ≤ 0.021, Table 2). For the FOR-T and FAT-C groups, the significant reduction in mNSS appeared since day 5 (one-way ANOVA, *P* = 0.000, EF = 0.612, Bonferroni *post hoc* tests *P* ≤ 0.011, Table 2) and day 4 (one-way ANOVA, *P* = 0.000, EF = 0.692, Bonferroni *post hoc* tests *P* ≤ 0.000, Table 2). The FAT-C group showed significantly lower mNSS compared with the CTRL group (day 3 to day 14, one-way ANCOVAs, *P* ≤ 0.003, EF ≥ 0.286, Bonferroni *post hoc* tests, *P* ≤ 0.005, Table 2) and the FOR-T group (day 4, day 8, day 10 to day 14, one-way ANCOVAs, *P* ≤ 0.003, EF ≥ 0.286, Bonferroni *post hoc* tests, *P* ≤ 0.042, Table 2). The FOR-T group showed significantly lower mNSS compared with the CTRL group (day 6 to day 14, one-way ANCOVAs, *P* ≤ 0.000, EF ≥ 0.413, Bonferroni *post hoc* tests, *P* ≤ 0.030, Table 2).

#### Motor Subscore

The motor subscore variations of the CTRL, FOR-T, and FAT-C groups during the rehabilitation are shown in Figure 7B. Two-way ANCOVA illustrated significant time point effect and group effect on motor subscore among the three groups (two-way ANCOVA, timepoint: *P* = 0.000, EF = 0.271, group: *P* = 0.000, EF = 0.118, Table 2). No significant motor subscore reduction was found in the CTRL group (one-way ANOVA, *P* = 0.120, EF = 0.126, Table 2). For the FOR-T group, a significant reduction in motor subscore was observed since day 6 (one-way ANOVA, *P* = 0.000, EF = 0.416, Bonferroni *post hoc* tests *P* ≤ 0.022, Table 2). For the FAT-C group, a significant motor score reduction was observed since day 7 (one-way ANOVA, *P* = 0.000, EF = 0.365, Bonferroni *post hoc* tests *P* ≤ 0.006, Table 2). The CTRL group showed significantly higher scores compared with the FAT-C group (day 11 to day 14, one-way ANCOVAs, *P* ≤ 0.005, EF ≥ 0.282, Bonferroni *post hoc* tests *P* ≤ 0.006, Table 2) and the FOR-T group (day 11 to day 14, one-way ANCOVAs, *P* ≤ 0.005, EF ≥ 0.282, Bonferroni *post hoc* tests, *P* ≤ 0.025, Table 2).

#### Beam Balance Subscore

The trends of beam balance subscore in the CTRL, FOR-T, and FAT-C groups from day 2 to day 14 are presented in Figure 7C. The CTRL group showed significantly lower baseline beam balance subscores compared with the FAT-C group (one-way ANOVA, *P* = 0.006, EF = 0.253, Bonferroni *post hoc* tests, *P* = 0.007) and the FOR-T group (one-way ANOVA, *P* = 0.006, EF = 0.253, Bonferroni *post hoc* tests, *P* = 0.034). Significant timepoint effect and group effect on beam balance subscores of the three groups (two-way ANCOVA, timepoint: *P* = 0.000, EF = 0.387, group: *P* = 0.000, EF = 0.308, Table 2) were observed. For the CTRL group, a significant reduction in beam balance subscore was found since day 9 (one-way ANOVA, *P* = 0.000, EF = 0.315, Bonferroni *post hoc* tests, *P* ≤ 0.004). The significant score reduction appeared since day 6 in the FOR-T group (one-way ANOVA, *P* = 0.000, EF = 0.417, Bonferroni *post hoc* tests *P* ≤ 0.009). The FAT-C group showed a significant reduction in the beam balance subscore since day 4 (one-way ANOVA, *P* = 0.000, EF = 0.486, Bonferroni *post hoc* tests, *P* ≤ 0.034, Table 2). The FAT-C group exhibited significantly lower scores compared with the CTRL group (day 3 to day 14, one-way ANCOVAs, *P* ≤ 0.023, EF ≥ 0.2, Bonferroni *post hoc* tests *P* ≤ 0.046, Table 2) and the FOR-T group (day 14, one-way ANCOVA, *P* = 0.002, EF = 0.334, with Bonferroni *post hoc* tests, *P* = 0.026, Table 2). The FOR-T group showed significantly lower scores compared with the CTRL group (day 6 to day 13, except day 9 and day 12, one-way ANCOVAs, *P* ≤ 0.001, EF ≥ 0.339, Bonferroni *post hoc* tests, *P* ≤ 0.045, Table 2).

#### Sensory Subscore

The variations of sensory subscore in the CTRL, FOR-T, and FAT-C groups during rehabilitation training are shown in Figure 7D. Two-way ANCOVA revealed significant time point effect and group effect on the three groups (two-way ANCOVA, timepoint: *P* = 0.000, EF = 0.084, group: *P* = 0.000, EF = 0.166, Table 2). The CTRL group showed a significant reduction in sensory subscore on day 9 and day 10 (one-way ANOVA, *P* = 0.007, EF = 0.188, Bonferroni *post hoc* tests *P* ≤ 0.019, Table 2). No significant sensory subscore reduction was found in FOR-T and FAT-C groups (one-way ANOVA, *P* > 0.05, Table 2). The FAT-C group exhibited significantly lower sensory subscores compared with the CTRL group (day 3 to day 5, day 7, day 11, day 13, and day 14, one-way ANCOVAs, *P* ≤ 0.034, EF ≥ 0.181, Bonferroni *post hoc* tests, P ≤ 0.036, Table 2) and the FOR-T group (day 10 to day 12, one-way ANCOVAs, *P* ≤ 0.027, EF ≥ 0.203, Bonferroni *post hoc* tests, *P* ≤ 0.042, Table 2).

### Symmetry Index

**Figure 8:**
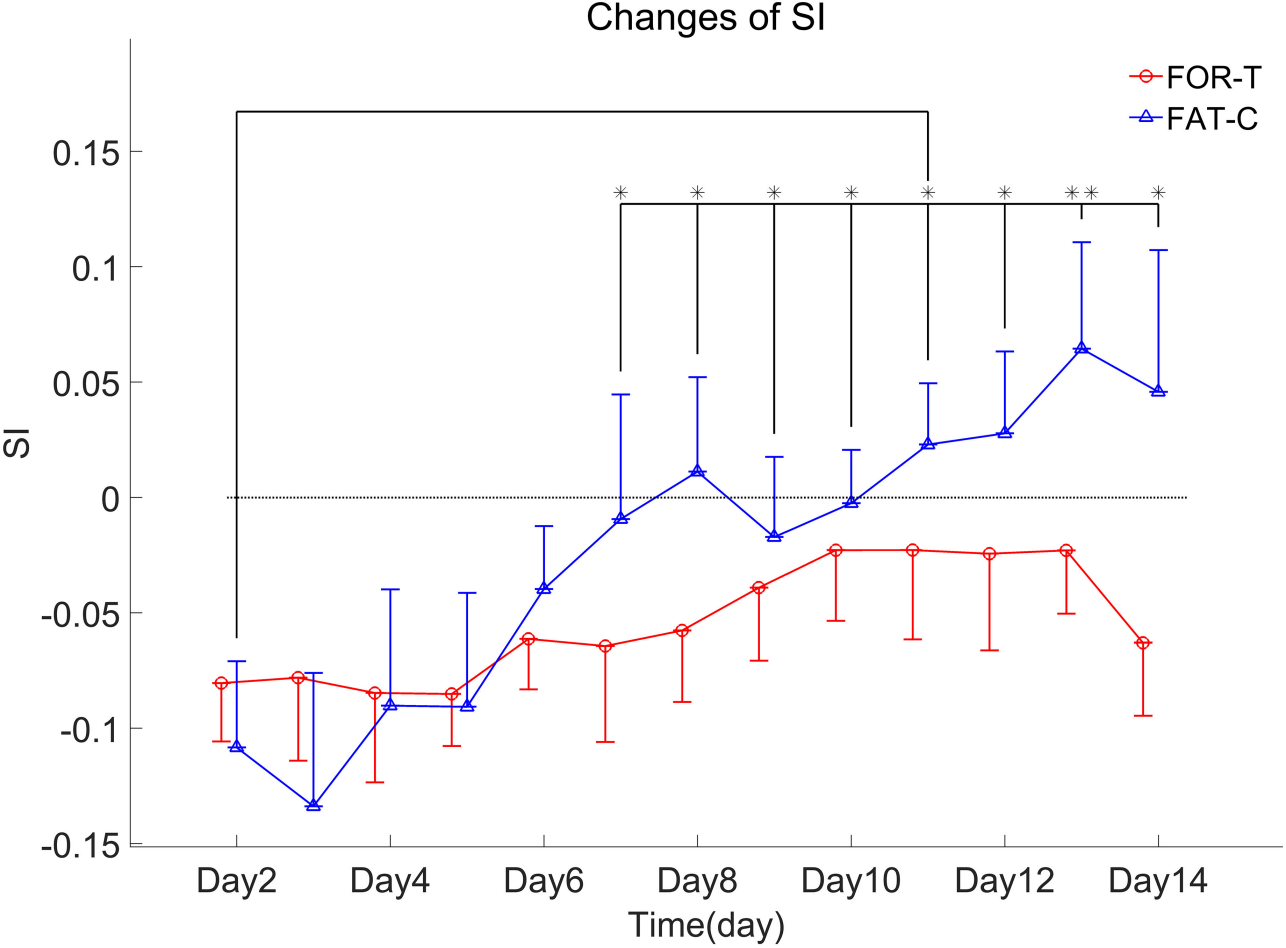
SI values of the FOR-T and FAT-C groups from day 2 to day 14 post-stroke were presented as mean value with 0.5*SD (error bar). Significant differences between day 2 and other time points are indicated by “*”. Significant levels were indicated as 1 superscript for <0.05, 2 superscripts for <0.005.

The trends of SI in the FOR-T and FAT-C groups during treadmill training are shown in Figure 8. Table 3 shows the two-way ANCOVA results of SI and predicted EFs, with independent factors of time point and group. The probabilities for the SI varying over the time in the FOR-T and FAT-C groups analyzed by one-way ANOVA are also included in Table 3. The results suggested a significant time point effect and group effect on the SI of the two groups (two-way ANCOVA, timepoint: *P* = 0.000, EF = 0.224, group: *P* = 0.001, EF = 0.065, Table 3). For intragroup analysis, the FAT-C group showed significantly higher SI compared with baseline SI since day 7 (*P* = 0.000, EF = 0.35, one-way ANOVA with Bonferroni *post hoc* tests *P* ≤ 0.046, Table 3). No significant SI variation was observed in the FOR-T group during the training (one-way ANOVA, *P* = 0.349, EF = 0.121, Table 3).

**Table 3:** Comparison of SI with respect to the independent factors of the time points and groups, SI on day 2 as covariate.

**Time point**	**SI, mean (95% confidence interval)**		**One-way ANCOVA, *P*-value (EF)**	**Two-way ANCOVA**, ***P*****-value (EF)**		
	**FOR-T**	**FAT-C**		**Time point**	**Group**	Time point*Group
Day 2	−0.0804 (−0.1432 to −0.0176)	−0.108 (−0.184 to −0.032)				
Day 3	−0.0781 (−0.1382 to −0.0179)	−0.134 (−0.198 to −0.070)	0.245 (0.217)	0.000*** (0.224)	0.001** (0.065)	0.246 (0.077)
Day 4	−0.0847 (−0.1443 to −0.0250)	−0.09 (−0.160 to −0.021)	0.862 (0.003)			
Day 5	−0.0799 (−0.1145 to −0.0452)	−0.091 (−0.151 to −0.031)	0.729 (0.010)			
Day 6	−0.0613 (−0.0979 to −0.0246)	−0.04 (−0.104 to 0.025)	0.233 (0.116)			
Day 7	−0.063 (−0.1161 to −0.0098)	−0.009 (−0.069 to 0.051)	0.207 (0.104)			
Day 8	−0.0576 (−0.1053 to −0.0099)	0.011 (−0.049 to 0.071)	0.063 (0.226)			
Day 9	−0.0391 (−0.0844 to 0.0063)	−0.017 (−0.077 to 0.043)	0.550 (0.024)			
Day 10	−0.0228 (−0.0667 to 0.0211)	−0.002 (−0.063 to 0.058)	0.542 (0.025)			
Day 11	−0.0228 (−0.0823 to 0.0368)	0.023 (−0.037 to 0.083)	0.249 (0.094)			
Day 12	−0.0243 (−0.0888 to 0.0402)	0.028 (−0.036 to 0.092)	0.238 (0.105)			
Day 13	−0.0229 (−0.0737 to 0.0279)	0.064 (−0.005 to 0.134)	0.051 (0.329)			
Day 14	−0.063 (−0.1214 to −0.0045)	0.046 (−0.030 to 0.122)	0.083 (0.297)			
One-way ANOVA, *P*-Value (EF)	0.349 (0.121)	0.000*** (0.35)				

Differences with statistical significance are marked with “*” (two-way ANCOVA, one-way ANCOVA, one-way ANOVA). Significant levels are indicated as 2 superscripts for <0.005, 3 superscripts for <0.001.

### Neurofilament-light Concentration in the Striatum

**Figure 9:**
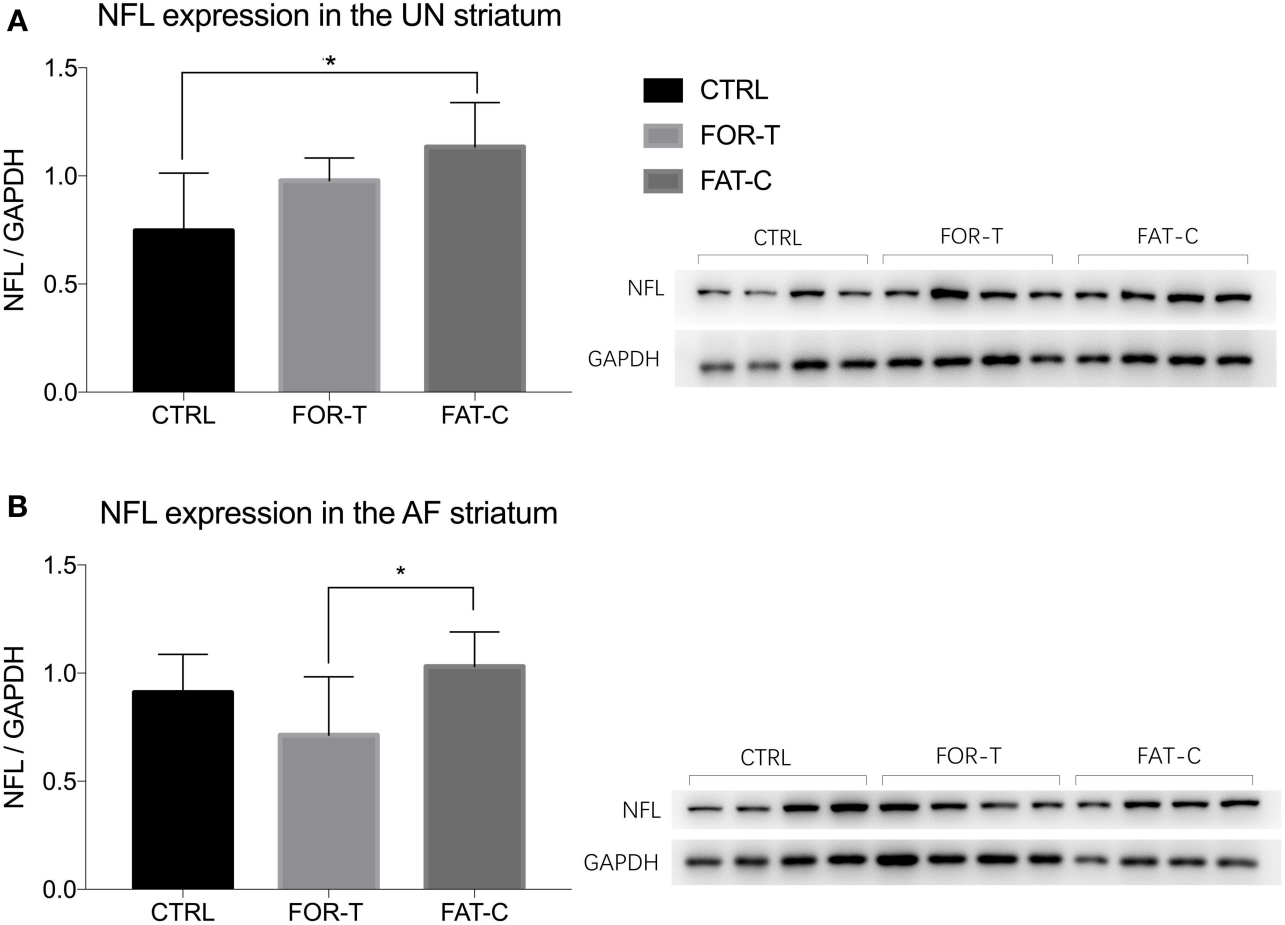
Concentration levels of NFL in the UN **(A)** and AF **(B)** striatum of the CTRL, FOR-T, and FAT-C groups measured by Western blot were presented as mean value with SD (error bar). Significant differences were indicated by “*”. Significant levels are indicated as 1 superscript for <0.05.

The relative protein levels of NFL in the AF and UN striatums of the CTRL, FOR-T, and FAT-C groups measured by Western blot are shown in Figure 9. Table 4 shows the mean values and 95% confidence intervals of the expression level of the NFL and one-way ANOVA probabilities with the predicted EFs on NFL concentration in the AF and UN striatums for the three groups. In the UN striatum, a significantly higher concentration was found in the FAT-C group compared with the CTRL group (one-way ANOVA, *P* = 0.005, EF = 0.412, Bonferroni *post hoc* tests *P* = 0.004, Table 4). In contrast, for the FOR-T group, no significant difference was found in the FAT-C and CTRL groups (one-way ANOVA, *P* = 0.005, EF = 0.412, Bonferroni *post hoc* tests *P* > 0.05, Table 4) in the UN side striatum. In the AF striatum, the FAT-C group showed a significantly higher NFL protein level compared with the FOR-T group (one-way ANOVA, *P* = 0.021, EF = 0.484, Bonferroni *post hoc* tests, *P* = 0.019, Table 4). Compared with the CTRL group, no significant difference was found in the FOR-T and FAT-C groups (one-way ANOVA, *P* = 0.021, EF = 0.484, Bonferroni *post hoc* tests, *P* > 0.05, Table 4).

**Table 4:** Comparison of NFL concentration on the UN and AF striatum.

**Tissue location**	**Group**	**NFL concentration, mean (95% confidence interval)**	**One-way ANOVA, *P*-value (EF)**
UN striatum	CTRL	0.747(0.579–0.914)	0.005*(0.412)
	FOR-T	0.978(0.834–1.123)	
	FAT-C	1.134(0.9997–1.270)	
AF striatum	CTRL	0.910(0.800–1.021)	0.021*(0.484)
	FOR-T	0.712(0.557–0.868)	
	FAT-C	1.034(0.878–1.189)	

Differences with statistical significance are marked with “*” (one-way ANOVA). Significant levels are indicated as, 1 superscript for <0.05.

## Discussion

In this study, the rehabilitation effects of fatigue-controlled training on post-stroke rehabilitation were investigated and evaluated by mNSS, EMG biomarker, and expression level of the NFL. We set up three rehabilitation training strategies, including no training, fatigue-controlled training, and forced training. The FAT-C group achieved a significantly lower mNSS and higher NFL level in the striatum than the CTRL and the FOR-T groups did. These results demonstrated that treadmill training with controlled muscular fatigue at a moderate level, i.e., 11% MPF drop in the AF side MG muscle, would lead to better post-stroke recovery in motor function and higher level neuroplasticity in the striatum compared with the traditional forced training.

### Electromyography-Based Real-Time Fatigue-Controlled Training

The fatigue-controlled platform could well manage the muscular fatigue level in the FAT-C group based on the monitoring of the EMG MPF drop rates during the running. The real-time fatiguing process in the MG muscles could be captured by the EMG MPF drop rates in both the FAT-C and FOR-T groups during the treadmill training (Figures 5A,B). The real-time MPF drop rates showed an increase followed by a plateau in the FOR-T group (Figure 5A), which suggested a maximum peripheral fatigue level maintained during the running. In contrast, the fatigue level of the FAT-C group was controlled and alleviated by the 3 min rest, as shown in the real-time MPF drop rate in Figure 5B, which demonstrated the fatigue level was controlled during training in the FAT-C group. The MPF drop rate in the AF hind limb of the FAT-C group was maintained at around 11% across sessions, and the significantly lower MPF drop rate was found in the FAT-C group compared with the FOR-T group (Figure 6). These cross-sessional MPF drop rates demonstrated that successful fatigue-controlled training was delivered to the FAT-C group.

To our knowledge, although individualized rehabilitative strategies have been attempted in previous studies (Pohl et al., 2002; Lau and Mak, 2011; Chen et al., 2015, 2019), this work was the first study to manage the individual treadmill training according to the real-time fatigue level obtained from the specific muscles of individuals. The fatigue-controlled training method could provide the real-time visualization and evaluation of the fatigue level through the EMG MPF drop rate. It could appropriately adjust the training or rest state for the rats based on the fatigue level. Our method was able to reduce the stress level and accomplished a more precise rehabilitation treatment. In addition, more thresholds of different muscles could be applied in the fatigue-controlled training platform to optimize the strategy for post-stroke rehabilitation in future studies.

### Different Fatigue Patterns Found in the Forced Training and Fatigue-Controlled Training Groups

The results that the FAT-C group outperformed the FOR-T group implied that individual training strategies possibly brought different effects on the fatigue of the hind limbs during the treadmill training and resulted in different rehabilitation mechanism adapted by the rats during the motor function recovery. Therefore, we further investigated the patterns of the MPF drop rate in the FAT-C and FOR-T groups.

Lower fatigue level was observed in the FAT-C group compared with the FOR-T group from day 2 to day 14 (Figure 6), and the decrease trends of training-induced fatigue were found in both the hind limbs of the FAT-C and FOR-T groups (Figure 6). For the FAT-C group, lower MPF drop rates were found in the AF hind limb compared with the UN hind limb (Figure 6) throughout the rehabilitation interventions. However, for the FOR-T group, lower MPF drop rates were found in the UN hind limb compared with the AF hind limb from day 2 to day 9 (Figure 6).

In the FAT-C group, the fatigue was controlled at a moderate level from day 2 to day 14. It was possible that the rats in the FAT-C group would prefer to use the UN hind limb when they were at a moderate fatigue level. In post-stroke moderate-intensity training, a less fatigue-related decrease in median frequency of EMG was also observed in humans at the paretic side compared with the non-paretic side during the same intensity voluntary contractions and locomotor activity (Riley and Bilodeau, 2002).

In contrast, in the FOR-T group, the MPF drop rate of the AF and UN hind limbs reached the maximum peripheral fatigue level because of overloaded usage. In high-intensity treadmill training, lower MPF drop rates were found in the UN hind limb compared with the AF hind limb (Li et al., 2011). This pattern was opposite to the situation found in the FAT-C group. However, lower MPF drop rates were observed in the AF hind limb compared with the UN hind limb from day 10 to day 14 in the FOR-T group. It might be related to increasing tolerance in the 30 min forced treadmill training, and the fatigue could be reduced by exercise-induced strengthening in muscle groups (Dobkin, 2008; Li et al., 2013).

### Fatigue-Controlled Training Group Showed Better Motor Function Recovery Compared With the Forced Training Group

The CTRL, FOR-T, and FAT-C groups showed significant motor function recovery at the end of rehabilitation interventions, but the FOR-T and FAT-C groups showed significantly better motor recovery compared with the CTRL group (Figure 7A). The FAT-C group showed significantly better motor function compared with the FOR-T group since day 10 (Figure 7A). Significant differences were observed in the late stage of rehabilitation intervention in the beam balance subscore (day 14, Figure 7C) and the sensory subscore (day 10 to day 12; day 13: *P* = 0.055; day 14: *P* = 0.079, Figure 7D). However, no significant difference was found between the FAT-C and FOR-T groups in the motor subscore (Figure 7B).

The motor function improvement in the CTRL group could be mainly due to the self-recovery after stroke, which was also observed in other studies (Takamatsu et al., 2010; Sun et al., 2014). The mortality rates in the three groups were 2/11 (CTRL), 0/11 (FOR-T), and 0/11 (FAT-C). The two rats in the CTRL group died in the early period post-stroke. The higher mortality rate implied the importance of post-stroke rehabilitation in the early stage. The behavior scores showed poorer motor function recovery at the early period of rehabilitation in the CTRL group compared with the FOR-T and FAT-C groups, which might account for the higher mortality rate in the CTRL group.

Treadmill training would benefit the motor function recovery, whereas the individual EMG MPF drop rate based fatigue-controlled training showed higher efficiency in motor recovery compared with the traditional forced training. Similar results were reported that the moderate or self-adapted rehabilitation training led to better motor function than high intensity forced training (Pohl et al., 2002; Ke et al., 2011; Lau and Mak, 2011; Li et al., 2013; Chen et al., 2019). Furthermore, recovery plateaus were found in the mNSS of CTRL (day 9 to day 14) and FOR-T (day 10 to day 14) groups but not in the FAT-C group. It might demonstrate that the maximum motor function recovery of the FAT-C group was higher than those of the CTRL and FOR-T groups. The CTRL and FOR-T groups had achieved their maximum motor function recovery, but there still would be a great potential for further motor recovery in the FAT-C group.

### Different Fatigue Patterns Led to the Differences in Both Symmetry Index and Motor Function Recovery

Both the FAT-C and FOR-T groups showed negative values of SI at the beginning of rehabilitation and increasing trends along with rehabilitation interventions (Figure 8). However, only the FAT-C group achieved significant balance improvement in the late stage of rehabilitation (day 7 to day 14, Figure 8). The negative values of SI represented more usage in the UN hind limb and the imbalance of hind limbs post-stroke. The motor deficits were also observed in the behavioral scores (Figure 7). The increasing trend of SI implied a more balanced usage of hind limbs and accompanied by the recovery of beam balance function (Figure 7C). The increase of SI paired with motor function recovery was also observed in previous studies (Li et al., 2011, 2013).

A significantly better beam balance subscore was found in the FAT-C group compared with the FOR-T group on day 14 (Figure 7C). The plateau of the beam balance subscore was observed in the FOR-T group since day 10, but no plateau of the beam balance subscore was found in the FAT-C group (Figure 7C). It implied a potential for further improvement on beam balance subscore in the FAT-C group. The beam balance score was related to the symmetry of limbs and trunk. The significant increase of the SI found in the FAT-C group (Figure 8) that indicated the improvement in beam balance in the FAT-C group was mainly related to the recovery of the AF hind limb, whereas the improvement of beam balance in the FOR-T group might be related to the compensation by other limbs or trunk. It was possibly caused by the different fatigue patterns and training strategies. In the FAT-C group, the less fatigue level was found in the AF hind limb compared with the UN hind limb (Figure 6). It was possible that moderate fatigue-controlled training promoted more usage on the AF hind limb and led to a better balance. Much more motor recovery was found in speed-dependent treadmill training compared with the steady-speed training in stroke survivors (Pohl et al., 2002; Lau and Mak, 2011). In the FOR-T group, the rats kept running at a maximum peripheral fatigue level (Figures 5A, 6) and could lead to the overloaded usage of the hind limbs. The extreme fatigue might cause the rat to fail to activate the AF hind limb and eventually lead to slower motor function recovery and poorer balance of hind limbs. It also implied that although both the treadmill training groups resulted in motor function recovery, the recovery mechanism might be different. The recovery mechanism of the FOR-T group might mainly be related to the compensation effects of the UN hind limb because overloaded treadmill training did not contribute significantly to the improvement of the balance of locomotion. In contrast, the recovery mechanism of moderate fatigue-controlled training might be related to the improvement of the motor function of AF hind limb and the balance of hind limbs.

### Different Training Strategies Caused the Different Neurofilament-Light Concentration and Resulted in Different Motor Function Recovery

Physical training in the subacute post-stroke period facilitated neuron plasticity and rewiring, but overloaded training-induced fatigue might minimize the plasticity. In this study, we found that the concentration of NFL in the UN striatum of the FAT-C group was significantly higher than that in the CTRL group (Figure 9A), and the concentration of NFL in the AF striatum of the FAT-C group was significantly higher than that in the FOR-T group (Figure 9B).

The increased concentration of NFL in the UN striatum (Figure 9A) and better motor function recovery (Figure 7) were found in the FOR-T and the FAT-C groups compared with the CTRL group. It might be related to the use-dependent dendritic growth and the compensation effects in the contralesional hemisphere caused by treadmill training. Treadmill training-related repetitive activation of cortical inputs could lead to long-term changes of synaptic plasticity in the related pathway (Zhang et al., 2013) and increase activation of cortico-subcortical networks (Luft et al., 2008), which were regarded as the cellular substrate for motor learning. The previous study also showed that the enhanced motor recovery was associated with significant increases in striatum volume, dendritic arbor in the contralesional striatum (Qin et al., 2014).

More importantly, we found a significantly higher NFL concentration in the FAT-C group compared with the FOR-T group in the AF striatum (Figure 9B). In contrast, a lower fatigue level of the AF hind limb (Figure 6) and better motor function (Figure 7) were found in the FAT-C group compared with the FOR-T group. The results indicated that physical training at a moderate fatigue level during the post-stroke subacute period would facilitate neuron plasticity and rewiring, but overloaded training-induced fatigue might harm the plasticity and lead to a poorer motor function recovery. Simultaneously, the excessive usage of AF hind limb would increase the stress and corticosterone secretion, which would reduce the protein level of the NFL and suppress the plasticity (Cereseto et al., 2006; Zhao et al., 2009). Overreliance on the AF forelimb after unilateral lesions of the forelimb representation area of the rat sensorimotor cortex led to an exaggeration of the initial cortical injury (Humm et al., 1999). So, rehabilitation training with controlled fatigue level in the AF hind limb would benefit the neuroplasticity and improve the efficiency of motor function recovery.

In this study, we measured the concentration of the NFL as a biomarker for post-stroke neuroplasticity. More neuroplasticity related indexes such as long-term depression and long-term potentiation will be included in our future studies to investigate the effects of fatigue-controlled rehabilitation.

## Conclusion

In this study, the EMG-based real-time fatigue-controlled training platform was established. Our results demonstrated that fatigue-controlled training was the most effective intervention in motor recovery compared to the forced training and the control groups. The EMG activation symmetry in the hind limbs also demonstrated that the treadmill training at a moderate fatigue level would facilitate the motor recovery of the AF hind limb and the balance between the hind limbs. This study also showed that fatigue-controlled training could upregulate the NFL level in the striatum and benefit neuroplasticity after stroke. We extended the understanding of the importance of fatigue-controlled training in rehabilitation after stroke. A training protocol that includes individual fatigue-controlled could be beneficial in both animal studies and clinical trials.

## Data Availability Statement

The raw data supporting the conclusions of this article will be made available by the authors, without undue reservation.

## Ethics Statement

The animal study was reviewed and approved by Animal Care Committee of Zhejiang University and the Animal Subjects Ethics Sub-committee, Hong Kong Polytechnic University.

## Author Contributions

YX contributed to the experiment design, data collection and analysis, and manuscript drafting. YY and HL contributed to the experiment design and data analysis. SN, YX, WP, and YZ contributed to the experiment design and manuscript editing. SZ and XH conceived the study and coordinated the whole project, including the experiment design, system design, and manuscript drafting. All authors contributed to the article and approved the submitted version.

## Conflict of Interest

The authors declare that the research was conducted in the absence of any commercial or financial relationships that could be construed as a potential conflict of interest.
